# Dynamics of Protein Phosphorylation during *Arabidopsis* Seed Germination

**DOI:** 10.3390/ijms23137059

**Published:** 2022-06-24

**Authors:** Emmanuel Baudouin, Juliette Puyaubert, Patrice Meimoun, Mélisande Blein-Nicolas, Marlène Davanture, Michel Zivy, Christophe Bailly

**Affiliations:** 1 Laboratoire de Biologie du Développement, UMR 7622, Institut de Biologie Paris-Seine (IBPS), Sorbonne Université, CNRS, F-75005 Paris, France; juliette.puyaubert@sorbonne-universite.fr (J.P.); patrice.meimoun@sorbonne-universite.fr (P.M.); christophe.bailly@sorbonne-universite.fr (C.B.); 2 PAPPSO, Génétique Quantitative et Evolution (GQE), Université Paris-Saclay, INRAE, CNRS, AgroParisTech, F-91190 Gif-sur-Yvette, France; melisande.blein-nicolas@inrae.fr (M.B.-N.); marlene.davanture@inrae.fr (M.D.); michel.zivy@inrae.fr (M.Z.)

**Keywords:** *Arabidopsis thaliana*, seeds, germination, dormancy, protein phosphorylation, protein kinases, protein phosphatases

## Abstract

Seed germination is critical for early plantlet development and is tightly controlled by environmental factors. Nevertheless, the signaling networks underlying germination control remain elusive. In this study, the remodeling of Arabidopsis seed phosphoproteome during imbibition was investigated using stable isotope dimethyl labeling and nanoLC-MS/MS analysis. Freshly harvested seeds were imbibed under dark or constant light to restrict or promote germination, respectively. For each light regime, phosphoproteins were extracted and identified from dry and imbibed (6 h, 16 h, and 24 h) seeds. A large repertoire of 10,244 phosphopeptides from 2546 phosphoproteins, including 110 protein kinases and key regulators of seed germination such as Delay Of Germination 1 (DOG1), was established. Most phosphoproteins were only identified in dry seeds. Early imbibition led to a similar massive downregulation in dormant and non-dormant seeds. After 24 h, 411 phosphoproteins were specifically identified in non-dormant seeds. Gene ontology analyses revealed their involvement in RNA and protein metabolism, transport, and signaling. In addition, 489 phosphopeptides were quantified, and 234 exhibited up or downregulation during imbibition. Interaction networks and motif analyses revealed their association with potential signaling modules involved in germination control. Our study provides evidence of a major role of phosphosignaling in the regulation of Arabidopsis seed germination.

## 1. Introduction

Seed germination is a major step in plant growth and development. It is critical for species competition and spreading capacity in ecosystems. In agrosystems, it eventually impacts crop growth and yield. To prevent unappropriated germination under environmental conditions that do not guarantee the establishment of a robust plantlet, seeds from temperate species are generally dormant at maturity. Dormancy is a physiological mechanism that blocks seed germination even under favorable conditions, and dormancy release is therefore required prior to germination [1]. A range of environmental (e.g., temperature, light, oxygen availability) and endogenous (e.g., hormonal) signals regulate these processes, and germination completion, i.e., the early emergence of embryo radicle from the seed envelope, can be achieved only when promoting mechanisms overcome inhibiting processes [2]. In that sense, the balance between the two antagonistic hormones abscisic acid (ABA) and gibberellins (GA), which inhibit and stimulate seed germination, respectively, promotes either dormancy (high ABA and low GA contents) or germination (low ABA and high GA contents) [3].

The transition from seed maturation to seed germination programs goes along with radical changes in seed metabolism [4]. In particular, seeds undergo dramatic modifications of protein patterns between the start of imbibition and the emergence of the radicle, as evidenced by proteomic analyses carried out in various species [5]. In this time lapse, protein synthesis essentially relies on the translation of mRNAs stored during seed maturation, and de novo gene transcription is not a requisite for germination completion [6]. Translation is, therefore, tightly regulated and selective, partly based on specific post-transcriptional modifications conferring unique features to the mRNAs to be translated or degraded [7]. Recent works further evidenced that the mRNAs translated in dormant and non-dormant seeds upon imbibition are different, which may condition the ability of seeds to germinate [8,9]. In addition, selective degradation of proteins is also required, which allows the removal of inhibitory factors. This is the case for the ABA Insensitive 5 (ABI5) transcription factor, a key component of the ABA signaling pathway, and for Repressor of Ga1-3-Like2 (RGL2), a member of DELLA proteins involved in GA signaling, that both participate in blocking germination [10,11].

Beyond the remodeling of protein patterns, reversible post-translational modifications of proteins in seeds have recently gained increasing attention [12]. In addition to other processes, e.g., oxidation, carbonylation, S-nitrosylation, or glycosylation, the reversible phosphorylation of proteins, that ultimately regulates protein activity, stability, localization, and/or protein/protein interaction, could play a major role in germination control. Indeed, key players of the regulatory network controlling seed germination are regulated by phosphorylation. For instance, the phosphorylation of ABI5 prevented its degradation, thereby promoting ABA response [13], and eight phosphosites that may participate in this process have been identified [14]. A similar mechanism might also regulate RGL2 stability. Indeed, substitutions mimicking amino acid phosphorylation impaired RGL2 degradation [15], and the phosphorylation of the rice DELLA OsSLR1 negatively regulated GA signaling [16]. Moreover, a series of protein kinases and phosphatases that play critical roles in the regulation of germination and essentially operate in ABA signaling have been identified [17]. For instance, three members of SNF1-related protein kinase 2 Subclass III, i.e., SNRK2.2/3/6, regulate positively the ABA response during seed dormancy and germination, at least partly via the phosphorylation of ABI5 [18]. Other classes of protein kinases, including mitogen-activated protein kinases, i.e., MPK6 and MPK8, and calcium-dependent kinases, i.e., CPK12, have also been involved in the regulation of seeds’ ability to germinate [19,20,21]. Conversely, the protein phosphatases 2Cs ABI1/2 and HAB1/2, which dephosphorylate and inactivate SNRK2s, inhibit ABA response in seeds [22,23].

So far, only a few studies have addressed the global modification of seed phosphoproteome in relation to seed germination. So far, the most comprehensive analyses have been carried out on cereals. Han and colleagues identified 413 phosphorylated proteins in dry and imbibed rice embryos [24]. Of these, 149 exhibited changes in phosphorylation level over imbibition duration (0–48 h), and the majority of them underwent a transient or sustained increase in phosphorylation level. Interestingly, the variation in protein abundance and protein phosphorylation level reported concerned distinct proteins and distinct functional classes, indicating that translational and post-translational regulations could be globally dissociated. Li and colleagues (2015) further identified 29 additional phosphoproteins isolated from rice embryo nuclei that underwent changes in phosphorylation level upon imbibition [25]. Differences associated with dormancy state have been reported recently in barley grains [26]. Although the abundance of 297 of the 395 phosphopeptides affected in freshly-harvested (dormant) and after-ripened (non-dormant) grains increased during imbibition, 90% of them were specific for either state, suggesting different remodeling of grain phosphoproteome correlated with germination capacity [26]. Moreover, ABA treatment provoked distinct phosphoproteome modifications in freshly-harvested and after-ripened grains, indicating the importance of phosphorylation for ABA signaling in seeds, but also the different responsiveness of dormant and non-dormant seeds to ABA [27]. Changes in seed phosphoproteome upon imbibition have also been reported in *Arabidopsis* [28]. In contrast to cereals, early and massive protein dephosphorylation affecting 238 of the 258 phosphoproteins identified has been reported [28]. Interestingly, the dephosphorylated proteins were essentially associated with dormancy, suggesting that the dephosphorylation of specific proteins might be a pre-requisite for germination [28]. These different reports, therefore, suggest the existence of different waves of protein phosphorylation/dephosphorylation that might control specific aspects of seed germination.

Temperature, nitrates, and light are critical environmental factors for regulating seeds’ capacity to germinate [29]. In Arabidopsis, light participates in the relief of dormancy, and its effect is particularly marked when dormancy deepness is low [30]. In the present study, we analyzed the dynamics of *Arabidopsis* seed phosphoproteome in freshly harvested seeds imbibed in darkness (thereafter referred to as DI seeds, in which germination is restricted) or under continuous light (referred to as LI seeds, in which germination is promoted).

## 2. Results

### 2.1. A Large Repertoire of Phosphoproteins Identified in Dry and Imbibed Arabidopsis Seeds

**Figure 1:**
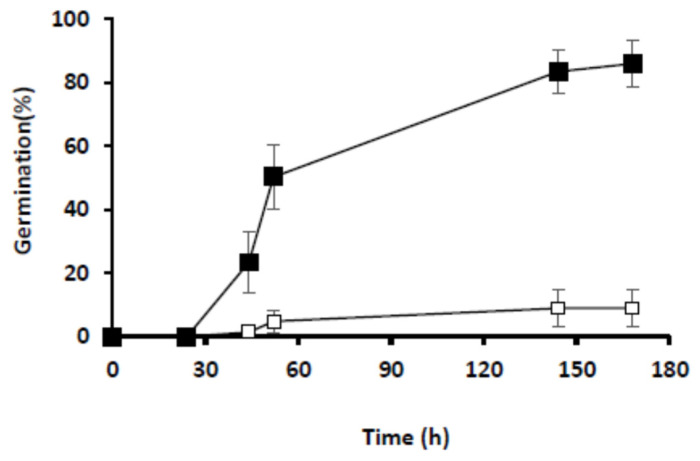
Germination of freshly-harvested Arabidopsis Col-0 seeds imbibed in darkness (open squares) or under continuous light (closed squares). Germination was scored at the indicated time. Values represent the mean S.E of three independent seed batches (150 seeds per batch).

The first objective of our study was to provide a comprehensive repertoire of the phosphoproteins present in *Arabidopsis* seeds during the achievement of germination. Proteins and phosphoproteins were extracted from dark-imbibed (DI) and light-imbibed (LI) seeds before (dry seeds) and after 6, 16, and 24 h of imbibition. As shown in Figure 1, these different time points precede visible germination that is not observed before 44 h for both conditions. Moreover, after 7 days, DI seeds had poorly germinated compared to LI seeds (8.9% and 86.1%), indicating that DI seeds remained essentially dormant (Figure 1).

**Figure 2:**
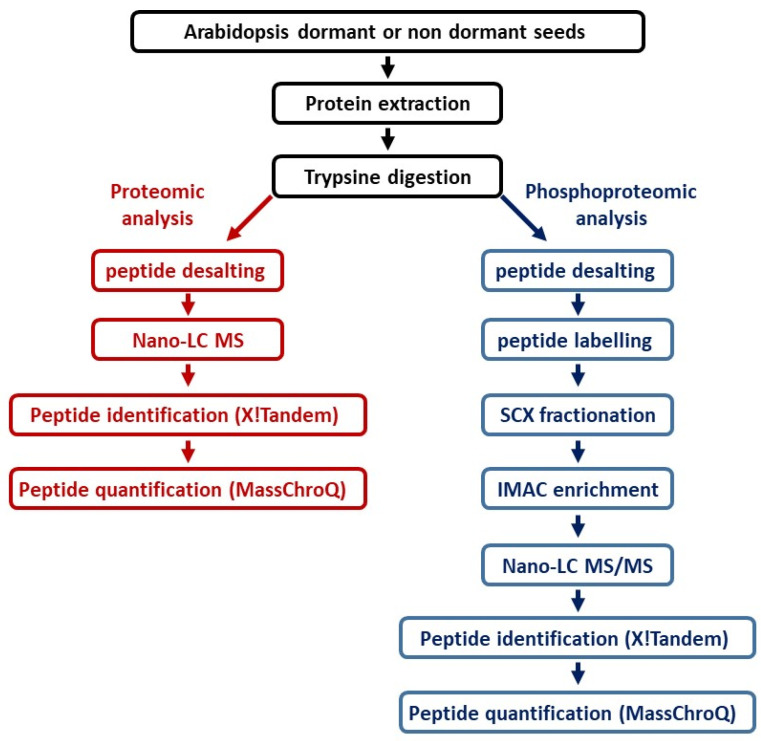
Overview of the workflow for seed protein and phosphoprotein identification.

Proteins extracted from DI and LI seeds were used to perform parallel large-scale proteomic and phosphoproteomic analyses, as summarized in Figure 2.

**Figure 3:**
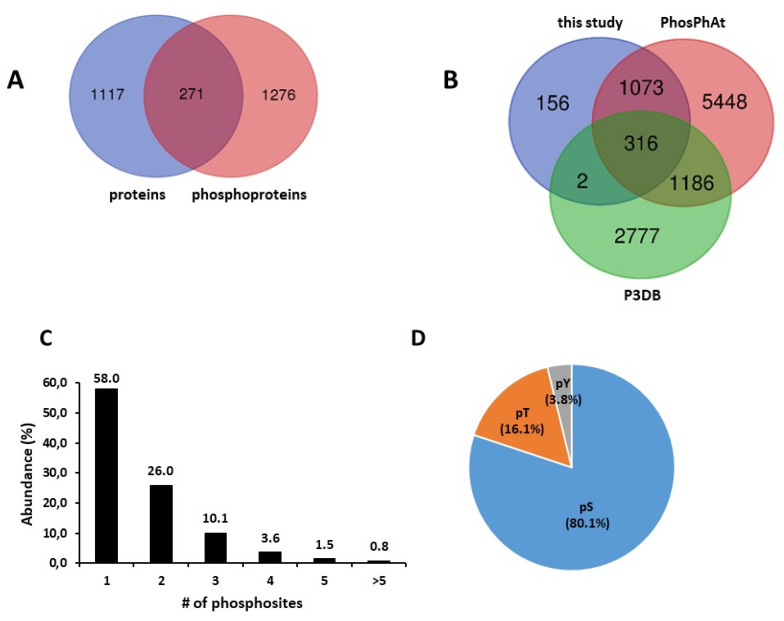
Overview of the seed proteome and phosphoproteome. (**A**) overlap of proteome and phosphoproteome. (**B**) overlap of identified phosphoproteins with known phosphoproteins from PhosPhAt 4.0 and P3DB databases. (**C**) distribution of phosphoproteins according to the number of phosphosites identified. (**D**) Relative abundance of serine, threonine, and tyrosine phosphosites.

We thereby identified 10,332 peptides from 1695 proteins, and 10,244 phosphopeptides from 2546 phosphoproteins present in at least 1 of the samples repeat analyzed (Tables S2 and S3, respectively). From these proteins and phosphoproteins, only the ones that could be identified in at least two of three biological repeats for one time-point × light regime condition were subsequently considered for analysis (1388 proteins and 1547 phosphoproteins, corresponding to 9792 and 8997 peptides, respectively; Supplemental Table S4). As shown in Figure 3A, only 19.5% of the proteins identified were common to protein and phosphoprotein repertoires. As phosphoproteome analysis was performed following enrichment of phosphorylated peptides prior to LC-MS/MS, it allowed the detection of low abundant phosphoproteins that could not be detected in proteome analysis. On the other hand, 10.1% of the phosphoproteins identified in seeds were not referenced in the phosphoprotein databases PhosPhat and P3DB (Figure 3B). The presence of a single phosphosite was the most frequent (58%; Figure 3C). Nevertheless, multiple sites could be identified for a single protein, with >5 phosphosites in 0.8% of the seed phosphoproteins (Figure 3C). The phosphoresidues were essentially Ser (80.1%), and phosphorylated Tyr residues were identified in only 3.8% of the phosphopeptides (Figure 3D).

**Figure 4:**
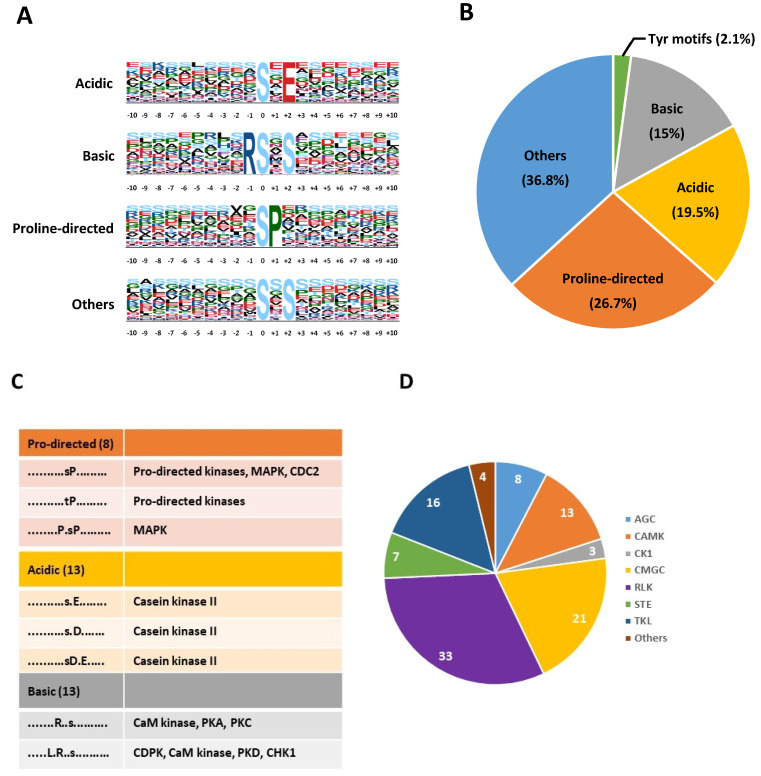
Identification of phosphorylation motifs enriched in seed phosphopeptides. (**A**) Overrepresented motifs identified by Motif-X software in pS/pT peptides. Full information is presented in Table S5. (**B**) Relative abundance of acidic, basic, Pro-directed, Tyr-containing, and other motifs in seed phosphopeptides. (**C**) Protein kinase classes preferentially targeting identified motifs. (**D**) Distribution of the protein kinases identified in seeds according to the iTAK database (http://bioinfo.bti.cornell.edu/tool/itak, accessed on 10 May 2022). Kinase families refer to KinBase nomenclature (http://kinase.com/web/current/kinbase/, accessed on 10 May 2022): AGC: PKA/PKG/PKC-related kinases; CAMK: Calcium/Calmodulin regulated kinases and structurally related families; CK1: Casein Kinase 1 group; CMGC: CDK/MAPK/GSK3/CLK group; RLK: receptor-like kinases; TKL: Tyr kinase-like group; STE: STE7/STE11/STE20 homologs. Full information is presented in Table S6.

To achieve additional insights into the characteristics of the phosphosites identified in seed proteins, we searched for conserved motifs surrounding the phosphoresidue using Motif-X software. Out of the 7296 peptides analyzed, the presence of a conserved motif was observed for 6292 (86.2%). The analysis led to the identification of 58 motifs significantly over-represented (47 for pSer, 8 for pThr, and 3 for pTyr, Table S5). For pSer/pThr, motifs could be further classified as acidic, basic, proline-directed, and others, according to the nature of the conserved residues within the motif (Figure 4A). As shown in Figure 4B and Table S5, acidic motifs characterized by the presence of Asp or Glu at the +1 or +2 position of the motif were identified in 19.5% of the 6292 phosphopeptides. Similarly, basic motifs with Lys or Arg at the −1, −2, or −3 position of the motif were found for 14.9% of the phosphopeptides (Figure 4A and Table S5). The most abundant class (26.7%) corresponded to Pro-directed motifs where a Pro residue is located at the position +1 (Figure 4B and Table S5). Finally, diverse additional motifs were found in 36.8% of the phosphopeptides. Several of the motifs identified have been proposed as preferentially targeted by diverse classes of protein kinases, such as MAP kinases for Pro-directed motifs, Casein Kinase II (CK-II) for acidic motifs, or calcium-regulated kinases (CaM kinases, CDPKs) for basic motifs (Figure 4C). As protein kinases are directly responsible for protein phosphorylation, the identity of the protein kinases present in seeds was extracted from the proteomic and phosphoproteomic repertoire (Table S6). From these data, 111 protein kinases could be identified and 105 classified according to the iTAK database [31]. Among them, 13 protein kinases related to CaMK/CDPK (11.7%) and 21 Pro-directed kinases (18.9%) fell into the CDK/MAPK/GSK3/CLK (CMGC) group that could participate in the phosphorylation of the specific motifs identified. Although no CK-II catalytic subunit was identified in our analysis, CK-II regulatory subunit B2 was detected in seed phosphoproteins (Table S6). In addition, STE (STE7/STE11/STE20 homologs) and TKL (Tyr kinase-like) groups gathered numerous upstream regulators of MAPK modules, i.e., MAP2Ks, MAP3Ks, and MAP4Ks, that might participate in kinase modules operating in seed biology (Table S6). Moreover, 21 protein phosphatases were identified, including the PP2C AHG1 and the MAPK phosphatase PHS1, that function in ABA signaling in seeds (Table S6).

**Figure 5:**
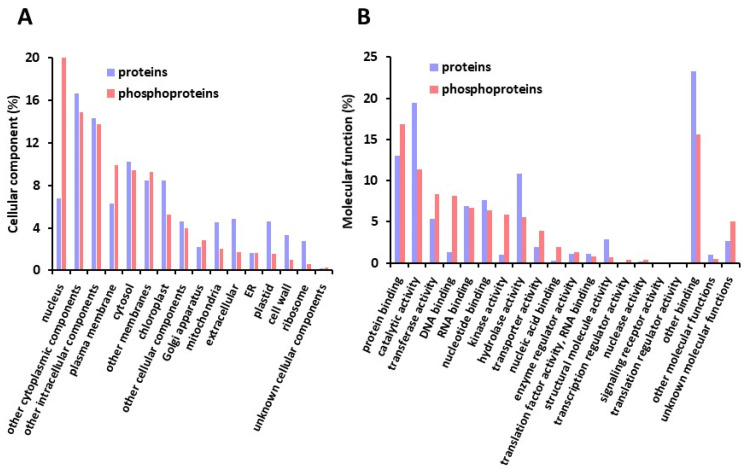

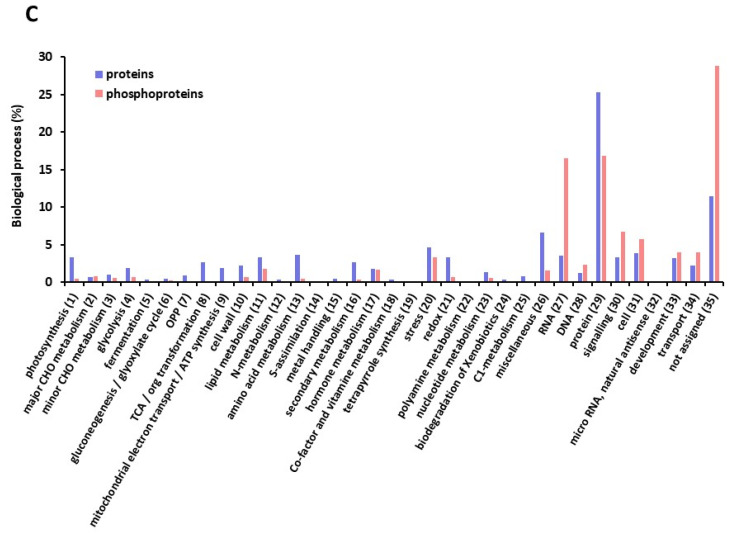
Functional classification of seed proteins and phosphoproteins. (**A**) cellular localization (according to TAIR GO classification). (**B**) molecular function (according to TAIR GO classification). (**C**) biological processes (according to MAPMAN using BAR interface).

The proteins and phosphoproteins identified were further classified according to their putative subcellular localization and molecular function (Figure 5A,B). Although (phospho) proteins originated from all the subcellular compartments, nuclear proteins were 3.2 fold more represented in the phosphoprotein repertoire (21.8% compared to 6.8% in the protein repertoire). In contrast, plastidial (including predicted chloroplastic proteins) and mitochondrial proteins represented a lower proportion of the phosphoproteins identified compared to that in the protein dataset (Figure 5A). In agreement with the number of nuclear phosphoproteins, DNA binding function was also more represented in phosphoproteins (8.2% compared to 1.3% for the protein dataset) (Figure 5B). Strikingly, proteins with kinase activity were also more abundant in the phosphoprotein dataset (Figure 5B). To achieve additional insights into the biological processes in which the seed proteins and phosphoproteins could operate, (phospho)proteins were classified according to MAPMAN functional classes. Classes referred to as “RNA” and “protein” gathered a large proportion of the (phospho)proteins identified (28.5 and 33.3% of the proteins and phosphoproteins, respectively). Noteworthy, the proportion of proteins compared to phosphoproteins was slightly higher in the “protein” class (1.5 fold), whereas that of phosphorylated proteins was strongly enhanced in the “RNA” class (4.7 fold) (Figure 5C). In addition, a globally higher representation of proteins compared to phosphoproteins was observed in classes 1–14, which cover diverse aspects of plant metabolism, although the proportion of proteins in each class was low (0.07–3.7%) (Figure 5C).

As a whole, the procedure carried out led to the identification of a large set of (phospho) proteins that could be further analyzed in the context of seed germination.

### 2.2. Modification of the Composition of Seed Phosphoproteome in Dark-Imbibed and Light-Imbibed Seeds

**Figure 6:**
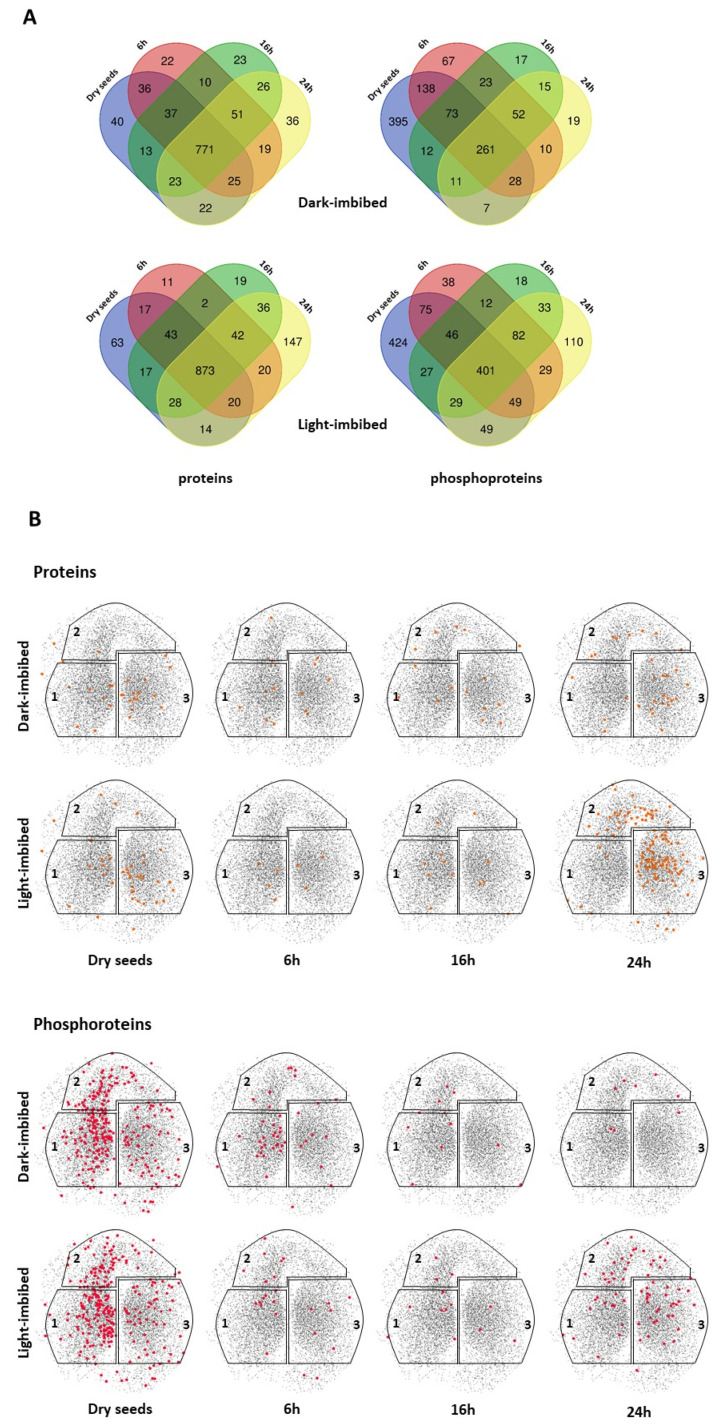
Modifications of DI and LI seed proteomes and phosphoproteomes during imbibition. (**A**) overlap of the proteins and phosphoproteins detected in dry and LI or DI seeds. (**B**) localization of proteins (orange dots) and phosphoproteins (red dots) specifically detected in each condition in the SeedNet network. SeedNet region 1 and 3 gather co-regulated genes/proteins associated with dormancy and germination, respectively. Genes/proteins with unspecific patterns gather in the network’s region 2.

In the first level of analysis, the proteins and phosphoproteins were compared on the basis of their presence/absence after different durations of imbibition in dark-imbibed (DI) and light-imbibed (LI) seeds (Figure 6 and Table S4). As differences in their relative abundance were not considered at this stage, only qualitative changes occurring in seed proteome and phosphoproteome in relation to the capacity of seeds to germinate are presented in this paragraph.

For DI and LI seed proteomes, the majority of the proteins were detected at all the time points (79.7% and 81.3%, in DI and LI seeds, respectively) and in both DI and LI seeds (76.5% and 68.8%, respectively) (Figure 6A). At the different time points analyzed, only a few (11 to 63) proteins were specifically detected, with the exception of LI seeds after 24 h of imbibition, for which 147 new proteins were identified (Figure 6A). To study the relationship of these proteins with the seed germination capacity, we identified their position on the topological model SeedNet representing dormancy and germination-related transcriptional interactions [32]. When the proteins identified as specific in the different samples were plotted in the SeedNet network, those specific of 24 h LI seeds gathered in region 3, which is associated with germination (Figure 6B). No specific pattern was observed for the other samples. In contrast, dramatic qualitative changes were observed in seed phosphoproteomes (Figure 6 and Table S4). Indeed, a large proportion of the phosphoproteins identified were detected only in dry seeds (42.7% and 38.5% in DI and LI seeds, respectively), indicating a massive and early dephosphorylation of proteins occurring during imbibition of both DI and LI seeds (Figure 6A and Table S4). Noteworthy, although the extent of dephosphorylation was similar in DI and LI seeds, the identity of dephosphorylated proteins only partially overlapped (241 of the 395 and 424 proteins dephosphorylated in DI and LI seeds, respectively; Table S4). Whereas very few newly phosphorylated proteins appeared in DI seeds after imbibition, 110 new phosphoproteins were detected in LI seeds after 24 h imbibition (Figure 6A and Table S4). When plotted in the SeedNet network, most phosphoproteins identified in dry seeds were located in region 1, which is associated with dormancy (Figure 6B). Moreover, newly phosphorylated proteins at 6, 16, and 24 h were essentially located in region 1 in DI seeds, whereas they were found in all 3 regions in LI seeds, especially after 24 h imbibition (Figure 6B).

**Figure 7:**
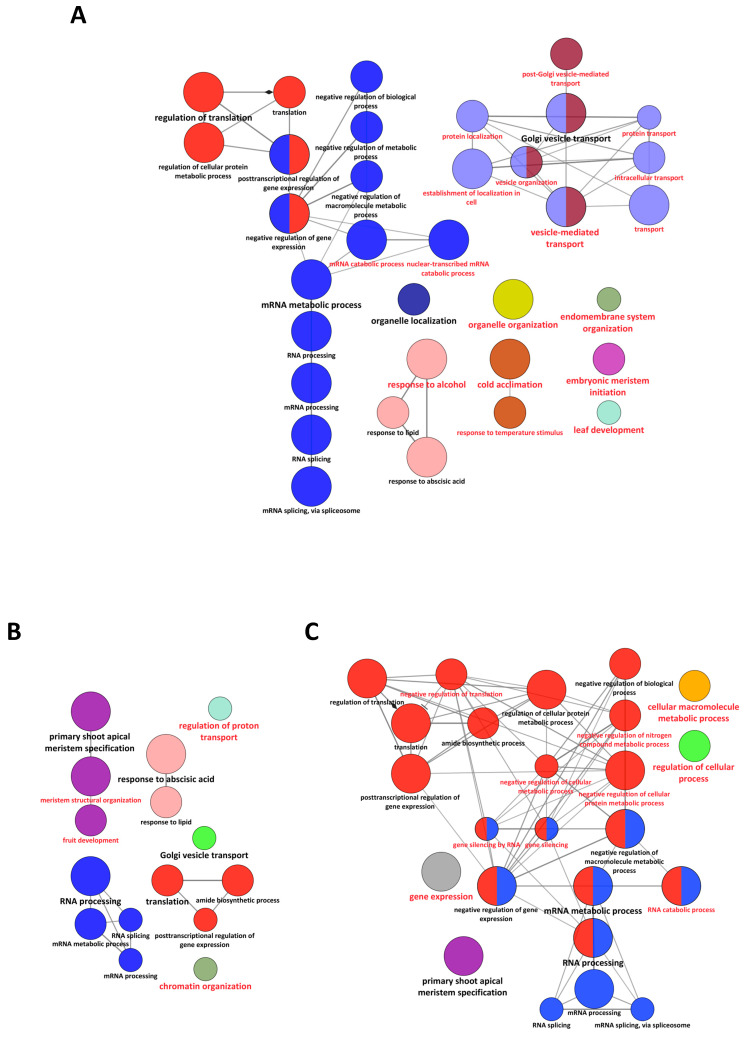
Overrepresented biological processes associated with phosphoproteins identified in dry (**A**) and 24 h DI seeds (**B**) and 24 h LI seeds (**C**). Enriched GO terms were identified using ClueGO application (pV < 0.01; hypergeometric test with Bonferroni pV correction). Node colors identify GO terms belonging to the same GO group. Red labels identify GO terms statistically overrepresented only in the condition analyzed. Detailed data are to be found in Table S7.

Overrepresented ontology terms in phosphoproteins from dry and 24 h DI and LI seeds were identified using the ClueGO application [33]. For dry seed phosphoproteins, 34 GO terms were overrepresented, half of them being specific for this condition (Figure 7A and Table S7). They referred to biological processes associated with cell organization and intracellular trafficking or to the response to temperature (Figure 7A). Although the majority of the 12 GO terms referring to mRNA metabolism were also overrepresented in imbibed seeds, 2 GO terms associated with mRNA catabolism were specific for dry seeds (Figure 7A). Only 15 overrepresented GO terms were identified for the 403 phosphoproteins from 24 h DI seeds, with 4 GO terms specific for this condition (Figure 7B and Table S7). Noteworthy, the GO term “response to abscisic acid” was shared with dry seeds but not with 24 h LI seeds (Figure 7B). In contrast, 24 overrepresented GO terms were identified in LI seed phosphoproteome (Figure 7C and Table S7). Most GO terms (14) referred to transcription or translation. Although several of them were also overrepresented in the dry seed phosphoproteome, specific terms such as “gene silencing” were only associated with this condition (Figure 7C).

**Figure 8:**
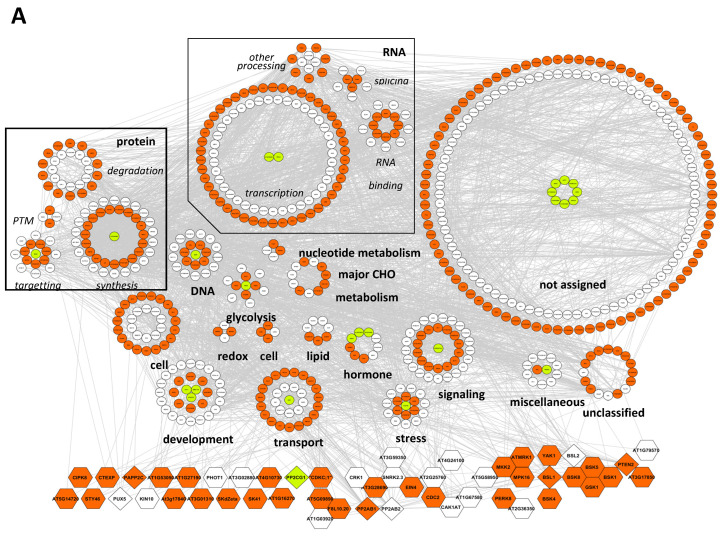

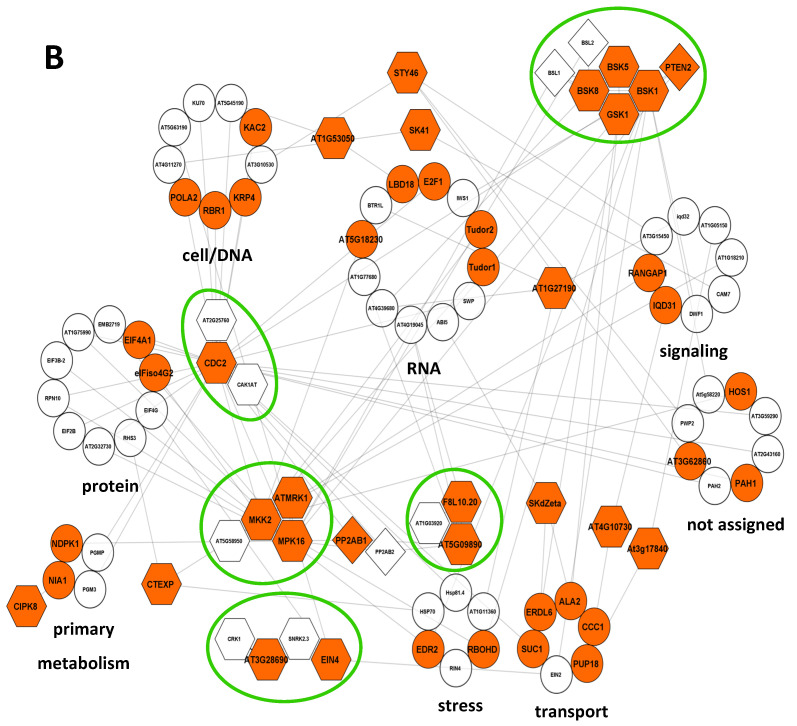
Association networks of phosphoproteins identified in 24 h-imbibed seeds. (**A**) Interactions of phosphoproteins identified in 24 h DI and LI seeds retrieved from STRING analysis (see Table S8 for complete data). Interactions with a confidence cut-off over 0.4 were selected and used for network construction in Cytoscape. The network was rooted onto identified kinases (hexagons) and phosphatases (diamonds). Phosphoproteins were grouped based on MAPMAN categorization. White symbols: phosphoproteins identified in DI and LI seeds; orange symbols: phosphoproteins identified only in LI seeds; green symbols: phosphoproteins identified only in DI seeds. (**B**) Simplified network including protein kinases specifically phosphorylated in 24 h LI seeds and their first-order interactors. Edges indicate direct interactions with kinases specifically phosphorylated in 24 h LI seeds. Phosphoproteins with enlarged labels were found specifically phosphorylated in LI seeds.

To further investigate functional relationships among phosphorylated proteins, a search for interactions within the 814 phosphoproteins identified in 24 h DI and LI seeds was performed using STRING 10.0 [34] (Figure 8A and Table S8). Associations were retrieved for 83.5% (680) of them with at least 1 partner (Table S8). As shown in Figure 8A, phosphoproteins specific for LI seeds and phosphoproteins present in both DI and LI seeds were identified in all the functional categories; nevertheless, LI-specific phosphoproteins were the most represented in “transport” (62%), “cell” (58.6%), and “transcription” (50.5%) categories. The association network included 43 protein kinases and 8 protein phosphatases, with 21 and 3 only identified in LI seeds, respectively (Figure 8A). Phosphorylation was reported as a mechanism of activation for several of these kinases (Table S9), possibly leading to the phosphorylation of downstream targets. To identify possible direct kinase/substrate modules, we extracted the kinases specifically phosphorylated in 24 h LI seeds together with their first order interactors from the network (Figure 8B). Out of the 73 direct interactors of these kinases, 26 were only found phosphorylated in LI seeds (Table S8). In addition, five putative kinase modules constituted of several interacting kinases could be identified, i.e., BSK1/5/8/-GSK1, AT5G58950-ATMRK1-MKK2-MPK16, EIN4-CRK1-AT3G28690-SnRK2.3, AT1G03920-AT5G09890-F8L10.20, and CDC2-CAK1AT-AT2G25780. The five modules presented several interactors, including LI-specific phosphoproteins (Figure 8B). For instance, 12 interactors were identified for CDC2, with 10 being specific for LI seeds, indicating an important function for this kinase in protein phosphorylation during germination. In contrast, only three proteins specifically phosphorylated in LI seeds were interactors for different kinases (RBOHD with MKK2 and BSK1, EDRL6 and ALA2 with SkDZeta and GSK1).

### 2.3. Modifications of the Abundance of Constitutive Seed Phosphoproteins in Dark-Imbibed and Light-Imbibed Seeds

**Figure 9:**
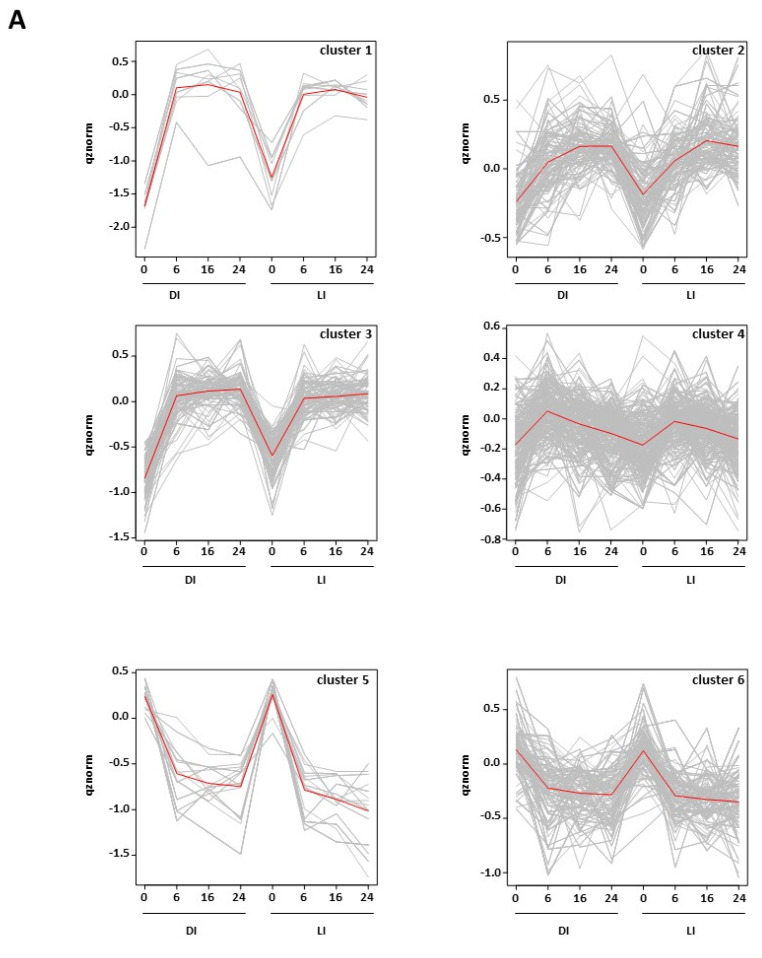

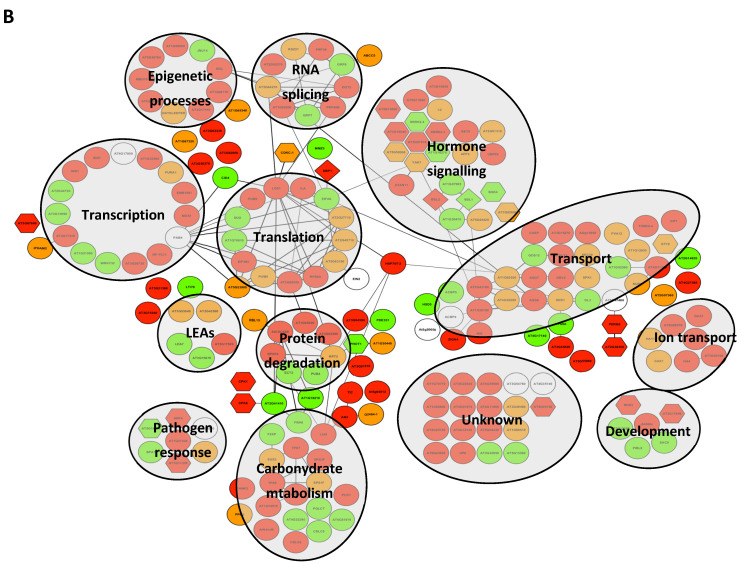

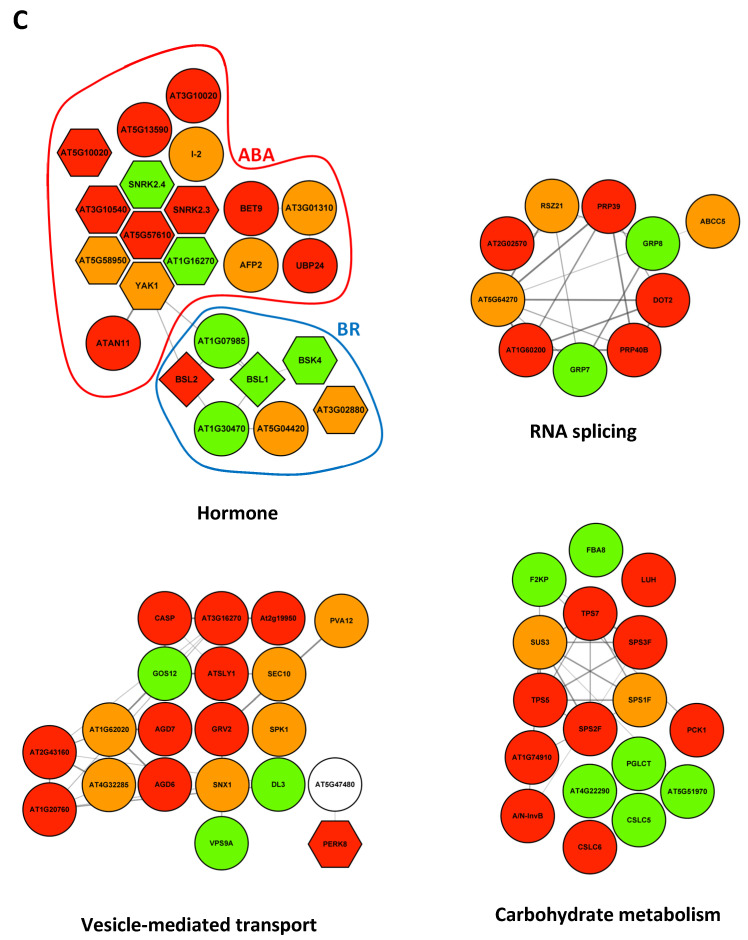
Quantitative changes in seed protein phosphorylation during imbibition. (**A**) Cluster analysis of the 489 phosphopeptides quantified in DI and LI seeds. (**B**) Association network of phosphoproteins undergoing changes in phosphorylation level during imbibition. Protein–protein interactions with a confidence cut-off over 0.4 were retrieved from the STRING application and used to construct a phosphoprotein network. Red and green nodes represent proteins with increased (cluster 1–3) or decreased (cluster 5–6) phosphorylation following imbibition, respectively. Orange nodes refer to proteins undergoing transient phosphorylation (cluster 4). Proteins exhibiting an opposite profile at different phosphosites are represented as white nodes. (**C**) Close-up of selected network regions corresponding to biological functions over-represented in the phosphoprotein dataset.

Although the majority of the phosphoproteins identified in this study were detected only at specific time points following imbibition (Table S4), a subset of proteins could be detected in almost all the conditions investigated, as shown in Figure 6A. We could quantify 489 phosphopeptides corresponding to 388 proteins identified in DI and LI seeds (Table S10). As shown in Figure 9A, different patterns of phosphorylation were identified by k-means clustering. Clusters 1, 2, and 3 corresponded to 186 phosphopeptides with increased abundance in imbibed seeds and exhibited slightly different kinetics; cluster 4 gathered 194 phosphopeptides undergoing a transient increase in abundance; clusters 5 and 6 corresponded to 109 phosphopeptides with decreased abundance in imbibed seeds. Out of these, 234 peptides corresponding to 210 proteins presented significant variations of abundance during imbibition (Table S10). In contrast to the qualitative analysis, no significant differences were found between DI and LI seed samples, and variations were only associated with imbibition duration (Table S10). As shown in Figure 9B, STRING interaction analysis led to an association network with a high degree of interaction (PPI enrichment = 2.2 × 10^−16^) and revealed biological connections among the phosphoproteins analyzed. A search for GO biological processes enrichment identified c.a. 80 overrepresented GO terms (Table S10). They refer to a variety of processes, including carbohydrate metabolism, regulation of transcription and translation, protein and ion transport, and responses to biotic and abiotic cues or hormone signaling (Table S9). In this latter process, 13 phosphoproteins were associated with the “response to abscisic acid” GO term and functioned in a variety of cellular processes, e.g., “protein metabolism” for EIF4G and RPN10, “vesicle-mediated transport” for GOS12 and DL3, or “RNA splicing” for GRP8 (Table S10). Conversely, additional phosphoproteins could be identified that, together with the aforementioned, constituted a cluster of 16 phosphoproteins related to ABA signaling (Figure 9C). With the exception of SnRK2.4 and AT1G16270, the phosphopeptides related to these proteins underwent a transient or sustained increase in abundance during imbibition. Half of them were kinases, including important regulators of ABA-dependent seed germination control such as SnRK2.3, YAK1, and the recently characterized RAF18 (AT1G16270) and RAF36 (AT5G58950). Through YAK1, the ABA signaling cluster interacted with two phosphoproteins (BSU-like 2 phosphatase (BSL2) and AT1G07985) belonging to a cluster of 7 phosphoproteins related to brassinosteroid (BR) signaling and that also included BSL1 phosphatase and BSK4 kinase (Figure 9C).

GO terms related to “mRNA splicing” were also overrepresented, and we could identify 9 phosphoproteins with a high degree of interaction that operated in this process (Figure 9C). For all these proteins, the corresponding phosphosites had been previously identified in vivo (Table S10). They included two pre-mRNA processing proteins (PRP39 and PRP40B) and the RSZ21 splicing factor that was phosphorylated during imbibition, and two Gly-rich proteins (GRP7 and GRP8), that participated in mRNA alternative splicing and were dephosphorylated during germination (Figure 9C). Interestingly, the phosphopeptides, the abundance of which increased during imbibition, all shared a pS/pT-P motif, suggesting that their regulation could be coordinated via the same kinase pathway(s) (Table S10). In contrast, GRP7 and GRP8, for which phosphopeptide abundance decreased, were phosphorylated on unrelated motifs (pS-G, pS-Q, pY-SG).

The abundance of the phosphopeptides of 14 proteins related to the “vesicle-mediated transport” process was increased/decreased during imbibition (Figure 9C). Golgi Snare 12 (GOS12) and dynamin-like 3 (DL3), involved in ER to Golgi vesicular transport and vesicle coating, respectively, shared a pS-P motif and the corresponding phosphopeptides were less abundant in imbibed seeds (Table S10). Among the seven proteins with enhanced phosphopeptide abundance, five (CASP, AGD6, AGD7, GRV2, and AT3G16270) shared a similar phosphorylation motif (pT/pSxE/D) targeted by Casein Kinase II (Table S10). Finally, a transient increase of the corresponding phosphopeptides was observed for five proteins, including the exocyst complex component SEC10 and the sorting nexin SNX1.

Finally, modifications of phosphopeptide abundance were also observed for enzymes of carbohydrate metabolism (Figure 9B). Indeed, seven proteins involved in disaccharide metabolism (Sucrose Synthase SUS3, Sucrose Phosphate Synthases SPS1/2/3, Trehalose-6-Phosphate Synthases TSP5/7, Alkaline/neutral Invertase A/N-InvB) presented phosphopeptide that was more abundant upon imbibition. In contrast, phosphopeptides for two enzymes of fructose metabolism, i.e., fructose 1,6-bisphophate 8 (FBA8) and fructose 6-phosphate 2 kinase/fructose 2,6-bisphophatase (F2KP), were less abundant. Enzymes and regulators of polysaccharide metabolism also underwent such changes during imbibition (Figure 9B and Table S10). They included CLSC5 and 6, two Cellulose Synthase-like enzymes involved in xyloglucan synthesis, pGclT, a plastidial glucose transporter involved in glucose remobilization following starch degradation, and Leunig-Homolog LUH, a regulator of seed mucilage extrusion. Contrarily to the former classes, phosphoproteins associated with carbohydrate metabolism were phosphorylated on a variety of motifs (Table S10).

## 3. Discussion

Reversible phosphorylation is the major regulatory mechanism operating in the signaling networks that control development and responses to environmental cues. In the present study, we provide an extensive view of *Arabidopsis* seed phosphoproteome and compare its modification in seeds imbibed in darkness or under continuous light.

Compared to other plant materials, seed phosphoproteome has been poorly investigated, and only a limited number of phosphoproteins and phosphopeptides have been identified, essentially in cereals [24,25,26,27,28,35]. This scarcity likely relies on the high content of unphosphorylated storage proteins in seeds. In our study, an enrichment of phosphopeptides by the IMAC technique was performed after an SCX fractionation step, lowering the complexity of each phosphopeptide fraction and optimizing LC-MS/MS analysis. The application of this approach to dry and dark- or light-imbibed seeds led to the identification of ~9000 phosphopeptides corresponding to 1547 phosphoproteins detected in at least one of the physiological conditions studied. The corresponding unphosphorylated proteins were rarely identified in the parallel proteome analysis (only 20% overlap), suggesting that phosphorylated proteins were essentially low-abundant proteins. Indeed, 69 phosphoproteins were transcription factors, and only one was identified in the proteome dataset. The general characteristics of phosphopeptides and phosphoproteins from *Arabidopsis* seeds, i.e., phosphoresidue distribution, sub-cellular compartment distribution, and biological function distribution (Figure 3D and Figure 4), resembled those previously extracted from *Arabidopsis* phosphoproteomics data meta-analyses [36]. Moreover, we identified 156 new phosphoproteins that were not referenced in PhosPhAt 4.0 and P3DB databases [37,38]. Nevertheless, most of these phosphoproteins (1462 out of 1547) were also reported recently as phosphorylated in seeds in a comprehensive atlas of *Arabidopsis* proteome [39]. The identified phosphoproteins in our study, therefore, constitute a pertinent set for further analysis of seed phosphoproteome remodeling in relation to germination.

The phosphoproteins identified were mainly present in dry seeds (79.6%) and largely associated with dormancy in the Seednet network [32]. Protein phosphorylation might, therefore, be an imprinting mark set during seed development and participating in maintaining seeds in a quiescent state. Noteworthy, this might be the case for DOG1, the major determinant of seed dormancy [40], that is phosphorylated in dry seeds. As previously reported [28], a massive dephosphorylation occurred in both DI and LI seeds between 6–16 h of imbibition. Although the extent of protein dephosphorylation was similar in DI and LI seeds, only 60% of the dephosphorylated proteins were the same in both conditions, suggesting that protein dephosphorylation could support different functions during the early stages of imbibition and might be involved in the dry-to-imbibed seed transition and in processes directly associated with germination capacity. Indeed, on the one hand, protein dephosphorylation might affect common mechanisms independently of the ability of seeds to germinate. In this view, it could participate in the general reactivation of metabolism consecutive to seed water content increase, dephosphorylation unlocking the blockade of metabolic enzymes that occured during seed desiccation. In our study, no enzyme of the primary metabolism was identified in the proteins dephosphorylated the same way in DI and LI seeds, and the association between dephosphorylation and metabolic reboot might, therefore, be indirect. On the other hand, 40% of the proteins undergoing dephosphorylation were specific for DI or LI seeds, suggesting that the dephosphorylation of particular proteins might be required for the non-germinating/germinating seed transition. Supporting this hypothesis, and in contrast to Xiang et al. (2016) [28], we identified several proteins related to ABA signaling in LI-specific dephosphorylated proteins, including the protein kinases RAF11 and CPK4 and the protein phosphatase PHS1. Mutant seeds of these three proteins present altered ABA sensitivity, and *raf11* seeds are poorly dormant [41,42,43]. It can therefore be proposed that the dephosphorylation of these proteins observed in LI seeds might participate in their inactivation, thereby allowing seed germination under continuous light. Finally, one has to consider that protein dephosphorylation not only potentially affects positively or negatively protein activity but also releases phosphate at the onset of the metabolic reboot. Although phytic acid is the major form of phosphate (P) storage in seeds [44], P stored on proteins could represent a valuable source of P for seeds during germination. In good agreement, abundant proteins, including storage proteins, have been found to be highly phosphorylated in various plant species [45,46,47]. We identified several abundant/storage proteins (oleosins, albumins, LEAs) that underwent rapid dephosphorylation and could participate in providing seeds with P.

In contrast to early time points, the phosphoprotein landscapes of DI and LI seeds strongly diverged after 24 h. On the one hand, most phosphorylated proteins detected in DI seeds were also found in LI seeds, and few proteins (32) were only phosphorylated in 24 h DI seeds. In this context, “response to ABA” was one of the few GO terms overrepresented in both dry seeds and DI seeds. The contribution of ABA to repress germination has been well and long-established, so the central role played by protein phosphorylation/dephosphorylation in ABA signaling has been to dry seeds on transition insured title and impinting mark [48]. Interestingly, three ABA-related phosphoproteins were specific for DI seeds, i.e., AFP1 and AFP2, two repressors of ABA signaling, and PP2CG1, a protein phosphatase 2C involved in ABA-dependent salt stress response [49,50]. AFP2 belongs to a repressor complex controlling ABA signaling in seeds and physically interacts with ABI5, leading to its degradation, and with AHG1, which itself inactivates ABI5 [51,52,53]. AFP2 phosphorylation could, therefore, modify its interaction with ABI5/AHG1, impair ABA signaling repression, and eventually favor germination repression. On the other hand, several hundreds of phosphoproteins specific for 24 h LI seeds were identified, including 110 proteins phosphorylated de novo, which suggests that the remodeling of the seed phosphorylation landscape is critical to promoting germination. The complex network of interactions among these phosphoproteins highlighted that the numerous functions could rely on phosphorylation-based regulation as its tight interlink with germination.

The regulation of mRNA metabolism has emerged as a major level of control of dormancy release and germination [54]. In addition to the modulation of gene transcription, specific alternative splicing of pre-mRNA, targeted mRNA storage or degradation, and selective mRNA translation have been reported to be correlated with seeds’ capacity to germinate and eventually trigger germination [9,55,56,57]. Strikingly, one-fourth of 411 phosphoproteins specific for 24 h LI seeds belong to the MAPMAN bin “RNA”, and 11 of the 27 GO terms overrepresented in this biological condition refer to RNA metabolism, suggesting that the modifications of phosphorylation status are central in post-transcriptional regulation. Nevertheless, whether protein phosphorylation might regulate RNA metabolism in a coordinated way to trigger germination is currently unknown, but several lines of evidence suggest that phosphorylation/dephosphorylation events might participate in the selectivity of mRNA post-transcriptional processes in non-germinating and germinating seeds. Firstly, dark-imbibition led to the dephosphorylation of proteins associated with mRNA processing (splicing), such as SR34, SR45, or RSZ32. An important role for alternative splicing in the light control of germination has been evidenced, and the phosphorylation of several pre-mRNA splicing factors has been reported [51,52,57,58,59]. In addition, Wang et al. (2013) showed that SR34, SR45, and RSZ32 splicing factors undergo dephosphorylation in response to ABA treatment. Although the outcome of such dephosphorylation is currently unclear, it could be part of an ABA-dependent pathway to restrict germination. In contrast, light-imbibition triggered the dephosphorylation of proteins involved in mRNA catabolism, such as VARICOSE (VCS), a major component of the 5′-to-3′ RNA degradation pathway. Together with the exonuclease XRN4, VCS participates in the selected degradation of mRNA during dormancy release and germination, and *vcs* mutants exhibit altered dormancy at harvest [56]. The phosphorylation of VCS by ABA-independent SnRK2s has been reported and participates in the regulation of mRNA decay under osmotic stress [53]. The outcome of the dephosphorylation of VCS and other components of mRNA catabolism is currently unknown, and its involvement in the control of germination needs to be investigated. Finally, we identified 81 RNA-binding proteins (RBPs) as phosphorylated in 24 h LI seeds, which represents c.a. 13% of the RBP proteome of seeds recently published [60]. Strikingly, 31 phosphorylated RBPs were specific to LI seeds, suggesting that their phosphorylation could be critical for the germination process. As evidenced for PABP, phosphorylation can modify RNA binding efficiency, so as RBP interactions with protein partners, including elongation factors, i.e., eIF4B, eIF(iso)4F, or eIF(iso)4G [61]. Among the LI-specific phospho-RBPs, we identified Tudor1/2, two RBPs associated with stress granules [62]. Tudor2 has been implicated in the modulation of *GA20ox3* mRNA level and thereby the regulation of seed germination [63]. Indeed, *tudor2* seeds present a higher dormancy at harvest [63]. In addition, the phosphorylation of Tudor2 mammalian homolog by c-Jun N-terminal kinase regulates its association with stress granules [64]. RBP phosphorylation might, therefore, play diverse and important functions for mRNA translation and storage in germinating seeds.

The reversible phosphorylation of proteins depends on the activity of hundreds of protein kinases and phosphatases that determine the dynamics of cell phosphoproteome. In this study, we identified 111 protein kinases and 21 protein phosphatases that may participate in the remodeling of seed phosphoproteome upon imbibition. Strikingly, 28 protein kinases (25%) were phosphorylated only in LI seeds which suggests that they may undergo specific regulation in this context. The impact of phosphorylation on the activity of these 28 kinases has only been reported for 5 of the 46 phosphosites identified (Table S9), and the functional significance of the others has yet to be investigated. In addition, a large proportion of protein kinases and phosphatases are not regulated by phosphorylation, and it is likely that the 111 kinases and 21 phosphatases identified are far from representing the whole set of active kinases and phosphatases present in dry and imbibed seeds. In good correlation with the large proportion (>25%) of seed phosphoproteins that exhibited an S/TP phosphosite, we identified several members of the MAP kinase family, e.g., MPK6, MPK11, MPK16, and MPK17 in seed phosphoproteome. Interestingly, the four MPKs were phosphorylated at the activating TEY/TDY site, suggesting that phosphorylation reflects kinase activation. Moreover, MPK11 phosphorylation was restricted to dry seeds, whereas MPK6 was phosphorylated in both DI and LI seeds, and MPK16 and MPK17 were identified as phosphorylated only in LI seeds. These different patterns suggest that these MPKs have different functions during seed germination. Whereas there is no information on a possible role of MPK11 in seeds, MPK6 has been associated with ABA signaling and seed dormancy [19]. MPK16 and MPK17 belong to the D class of MPKs, the function of which is currently poorly investigated. Nevertheless, preliminary data suggest that *mpk17* seeds germinate less at harvest than wild-type seeds [65]. Another member of the D class, MPK8, has also been recently characterized as a positive regulator of dormancy release and germination [21]. Future investigations are now required to unravel the possible functions and targets of MPK16/17 in germinating seeds. As previously reported in germinating rice grains [24], several kinases and phosphatases (BSK1/4/5/8, GSK1, BSL1/2) involved in brassinosteroid (BR) signaling were phosphorylated in imbibed *Arabidopsis* seeds. Interestingly, the phosphorylation of the protein kinases BSK1/5/8 and GSK1 was restricted to LI seeds. The three BSKs were phosphorylated on a conserved residue corresponding to S230 in BSK1. BSK1 is phosphorylated at S230 by the BR receptor BRI1, thereby activating BR responses [66]. BR are positive regulators of seed germination and have recently been ascribed a major function in seed-to-seedling transition [67]. Moreover, *bsk5* seeds exhibit delayed germination and a higher sensitivity towards ABA [68]. In the interaction networks, the BSK1/5/8 module was associated with the NADPH Oxidase RBOHD that was also specifically phosphorylated in LI seeds. Interestingly, *rbohD* mutant seeds exhibit a high dormancy at harvest, which is poorly released by after-ripening [69]. Although RBOHD is regulated by phosphorylation at multiple sites and by multiple kinases [70], most of the phosphosites identified in seeds (S8, S26, S769) have not been associated with any kinase so far and may, therefore, be targeted by BSKs. BSK phosphorylation of RBOHD might participate in the regulation of ROS production in seeds that are required for efficient germination [69]. This possible link between BR signaling and ROS signaling in seeds will require further investigation.

Light is a major signal controlling seed germination, and many factors involved in light-regulation of these processes have been identified [71]. In accordance with the experimental setting used in this study, i.e., absence or presence of light during seed imbibition, several proteins, i.e., SOMNUS (SOM), LEUNIG_HOMOLOG (LUH), LUX ARRYTHMO/PHYTOCLOCK 1 (LUX/PCL1), and PICKLE (PKL), involved in light control of germination were identified as phosphorylated. SOM and LUH phosphorylation was observed only in dry seeds in contrast with LUX/PLC1 and PKL, which were phosphorylated only in LI seeds after 24 h. SOM and LUH negatively regulated seed germination via the transcriptional regulation of ABA and GA metabolic genes [72,73]. *SOM* is repressed at the transcriptional level upon light exposure [72]. Moreover, the phosphorylation of LUH has recently been reported in sugarcane callus and is associated with embryogenic competency [74]. SOM and LUH dephosphorylation at the early stages of imbibition might be a new mechanism to control their stability and/or their biological activity. In contrast, LUX/PCL1 and PKL promote seed germination in response to light [75,76]. LUX is part of the so-called evening complex and participates in the repression of *DOG1* expression via the recruitment of PKL, a chromatin-remodeling factor, to the *DOG1* regulatory region [76]. The phosphorylation of LUX and/or PKL might, therefore, regulate their association and/or interaction with the DOG1 regulatory region and promote dormancy alleviation. Future work will help establish how the phosphorylation of SOM, LUH, LUX, and PKL participates in light-regulated germination and whether this post-translation regulation can be conserved in other light-dependent processes.

## 4. Materials and Methods

### 4.1. Seed Material

*Arabidopsis thaliana* ecotype Col-0 WT seeds were produced, harvested, and stored as previously described [69]. Three seed batches corresponding to independent production cycles were used for the study.

### 4.2. Germination Assays

Germination tests (150 seeds per freshly-harvested seed batch) were performed in growth chambers at 25 °C [9], in the darkness or under continuous light (3500 lux), as described. Germination was scored daily, according to radicle emergence through the testa.

For (phospho) protein analyses, 30 mg (c.a. 1500 seeds) of freshly-harvested seeds were sown for each time point × seed batch × light regime condition and harvested after 0, 6, 16, and 24 h of imbibition by freezing in liquid N_2_.

### 4.3. Protein Extraction and In-Solution Digestion

Seed samples were ground in liquid nitrogen and resuspended in 10 mL of extraction buffer (0.5 M Tris-HCl, 0.7 M saccharose, 50 mM ethylene diamine tetra acetic acid (EDTA), 0.1 M KCl, 10 mM Thiourea, 2 mM phenylmethylsulfonyl fluoride (PMSF), 2% (*v*/*v*) β-mercaptoethanol) by rapid vortexing at 4 °C. Proteins were extracted by the addition of 10 mL of buffered phenol solution (Aquaphenol^TM^ pH 8, MP Biomedicals, Illkirch, France) and strong shaking for 30 min at 4 °C. After centrifugation (12,000× *g*, 30 min), the phenolic phase was collected, supplemented with 10 mL of extraction buffer, and shaken for 30 min. After centrifugation (12,000× *g*, 30 min), the phenolic phase was supplemented with 5 volumes of 0.1 M ammonium acetate (in methanol) and incubated overnight at −20 °C. After centrifugation (10,000× *g*, 30 min), protein pellets were successively rinsed with methanol, then acetone, rapidly dried, and stored at −80 °C.

Protein pellets were solubilized in 250 µL of solubilization buffer (6 M urea, 2 M thiourea, 10 mM DTT, 30 mM Tris-HCl pH 8.8, 0.1% Progenta Zwitterionic Acid Labile Surfactant I). Protein concentration was determined using the 2-D Quant-kit (GE Healthcare, Cleveland, OH, USA) with bovine serum albumin (BSA) as standard. For the phosphoproteomic approach, 12 internal standards were prepared by pooling 42 µg of each of the 24 protein extracts. For each sample and each standard, 1 mg proteins were alkylated with iodoacetamide at 40 mM final concentration for 45 min in the dark. The samples and standards were then diluted to 1 M urea by adding 50 mM ammonium bicarbonate. Protein digestion (sequencing grade modified trypsin, Promega, Madison, WI, USA) was performed at an enzyme/substrate ratio of 1:50 (*w*/*w*) during overnight incubation at 37 °C and stopped by adding trifluoroacetic acid at 0.6% (*v*/*v*) final concentration.

For the label-free shotgun approach, 40 µg proteins were digested as above and desalt using C18 solid-phase extraction (SPE) cartridges (strata XL 100 µm, Phenomenex, Torrance, CA, USA), dried, and kept at −20 °C until nanoLC-MS/MS analysis.

### 4.4. Stable Isotope Dimethyl Labeling for Phosphoproteomic Approach

Digests were spin-dried and resuspended in 1 mL of 5% formic acid (*v*/*v*). Stable isotope dimethyl labeling was performed according to the on-column procedure described by [77] using formaldehyde-CH_2_O (light labeling), formaldehyde-CD_2_O (intermediate labeling), or formaldehyde-^13^CD_2_O (heavy labeling). Each digest was loaded on a separate SepPak C18 cartridge column (3 mL; Waters, Milford, MA, USA) and washed with 0.6% acetic acid (*v*/*v*). SepPak columns were flushed 5 times with 1 mL of 1 of the 3 labeling reagents (50 mM sodium phosphate buffer pH 7.5 containing 30 mM NaBH_3_CN and 0.2% CH_2_O or CD_2_O (*v*/*v*) for light or intermediate labeling, respectively, or 30 mM NaBD_3_CN and 0.2% ^13^CD_2_O (*v*/*v*) for heavy labeling). SepPak columns were washed with 2 mL of 0.6% acetic acid (*v*/*v*). Labeled peptides were eluted with 500 µL of 0.6% acetic acid (*v*/*v*) and 80% acetonitrile (ACN, *v*/*v*). The 24 samples were labeled with the intermediate and heavy isotopes, whereas the internal standard was labeled with the light isotope (Table S1). In the end, 12 triplexes were produced, each resulting from a mix of 2 samples (one heavy and one inter-labeled) and an internal standard in a 1:1:1 abundance ratio.

### 4.5. Peptide Fractionation Using Strong Cation Exchange Chromatography (SCX)

The 12 triplexes were spin-dried and reconstituted in 500 µL solvent A (30% ACN (*v*/*v*), 0.5% formic acid (*v*/*v*), pH 3). SCX was performed at 200 µL/min using Zorbax BioSCX-Series II columns (0.8-mm inner diameter x 50-mm length; 3.5 mm particle size) and a Famos autosampler (LC Packings, Amsterdam, The Netherlands). After sample loading, the first 20 min were run isocratically at 100% solvent A, followed by an increasing pH gradient using solvent B (30% ACN (*v*/*v*), 0.5% formic acid (*v*/*v*), 540 mM ammonium formate, pH 5). Twelve SCX fractions for each triplex were automatically collected using an on-line Probot system (LC Packings).

### 4.6. Enrichment of Phosphopeptides Using Immobilized Metal Ion Affinity Chromatography (IMAC)

The 144 SCX fractions were spin-dried and resuspended in 300 µL solvent C (250 mM acetic acid, 30% ACN (*v*/*v*)). Peptides were gently mixed with 80 µL Phos-Select iron affinity gel (Sigma-Aldrich, St. Louis, MI, USA) and incubated at room temperature for 1 h using a tube rotator. The mixture was transferred into SigmaPrep spin columns (Sigma-Aldrich) and rinsed twice with 200 µL solvent C, then with 200 µL double distilled water. The bound phosphopeptides were eluted with 60 µL solvent D (400 mM NH_4_OH, 30% ACN) by centrifugation at 8200× *g*. Eluted phosphopeptides were spin-dried and kept at −20 °C until LC-MS/MS analysis.

### 4.7. nanoLC-MS/MS Analysis

On-line liquid chromatography was performed on a NanoLC-Ultra system (Eksigent, Dublin, CA, USA). A 4 µL sample was loaded at 7.5 µL min^−1^ on a precolumn cartridge (stationary phase: Biosphère C18, particles of 5 µm; column: 360/100 µm i.d., 2 cm length; Nanoseparations) and desalted with 0.1% formic acid in water. After 3 min, the precolumn cartridge was connected to the separating Biosphère C18 column (stationary phase: Biosphère C18, particles of 3 µm; column 360/75 µm i.d., 30 cm length; Nanoseparations). Buffers were 0.1% formic acid in water (solvent E) and 0.1% formic acid in ACN (solvent F). Peptide separation was achieved using a linear gradient from 5 to 95% of solvent F at 300 nL min^−1^ during 75 min for phosphopeptides enrichment and 110 min for label-free shotgun proteome. Eluted peptides were analyzed with a Q-Exactive mass spectrometer (Thermo Scientific, Courtaboeuf, France) using a nano-electrospray interface. Ionization was performed with a liquid junction and an uncoated capillary probe (10 µm i.d.; New Objective, Woburn, MA, USA). Peptide ions were analyzed using Xcalibur 2.3 with the following data-dependent acquisition steps: (1) full MS scan on a 400 to 1400 range of mass-to-charge ratio (*m*/*z*) with a resolution of 70,000 and (2) MS/MS (normalized collision energy: 27%; resolution: 17,500). Step 2 was repeated for the 8 major ions detected in step 1. Dynamic exclusion was set to 40 s.

### 4.8. Identification of Proteins and Phosphopeptides

Xcalibur raw data were transformed to mzXML open-source format and centroided using the msconvert software in the ProteoWizard 3.0.7069 package [78].

Protein identification was performed using the X!Tandem Piledriver 2015.04.01 (http://www.thegpm.org/TANDEM, accessed on 10 May 2022)) by querying MS/MS data against the TAIR 10 (http://www.arabidopsis.org/, accessed on 10 May 2022) protein database together with a standard contaminant database. The following parameters were used: one missed trypsin cleavage allowed, cys carboxyamidomethylation, light, intermediate, and heavy dimethylation of peptide N-termini and lysine residues were set as static modifications, while Met oxidation and phosphorylation of tyrosine, serine, or threonine residues were set as variable modifications. Precursor mass tolerance was 10 ppm, and fragment mass tolerance was 0.02 Da.

Identified proteins were filtered and grouped using X!TandemPipeline 3.3.4 (pappso.inra.fr/bioinfo/xtandempipeline/) [79] according to (i) a minimum of two different peptides required with an E value smaller than 0.01, (ii) a protein E value (calculated as the product of unique peptide E values) smaller than 10^−5^. Criteria used for phosphopeptides identification were (i) one peptide identified with an E-value smaller than 0.001 and (ii) a protein E-value (product of unique peptide E-values) smaller than 10^−3^.

### 4.9. Quantification of Peptides and Phosphopeptides

Relative quantification of peptides was performed using MassChroQ version 2.2.2 [80] by extracting ion chromatograms (XICs) of all identified peptides within a 10 ppm window and by integrating the area of the XIC peak at their corresponding retention time. Parameters for peak detection threshold on max was 50,000 and 30,000 on min. Mean filter half edge set 1, minmax half edge set 3, and maxmin half edge set 2.

LC-MS/MS chromatogram alignment was performed by using common MS/MS identifications as landmarks to evaluate retention time deviations along with the chromatographic profiles. For phosphopeptide enriched fractions, alignments were performed with 12 groups of LC-MS/MS runs originating from similar fractions and 1 group for the shotgun approach.

The mass spectrometry proteomics data have been deposited to the ProteomeXchange Consortium via the PRIDE [81] partner repository with the dataset identifier PXD033347.

### 4.10. Data Analysis

For qualitative analyses, proteins and phosphoproteins that were identified in two of the three replicates of at least one time-point × light regime condition were considered.

For quantitative analyses, two-way ANOVA was performed after normalization of phosphopeptide relative quantities. Phosphopeptides with *p*-value < 0.05 and FRD < 0.05 were considered as undergoing significant change.

Phosphorylation motif analysis was performed with the Motif-X application (https://motif-x.med.harvard.edu/; accessed on 6 February 2017) using 21-mer peptides centered on pS/pT/pY residues and a cut-off of *p*-value < 10^−6^ and occurrence >20 and >10 for pS/pT and pY, respectively. Motif classification and putative kinase association were retrieved with PhosphoMotifFinder (http://hprd.org/PhosphoMotif_finder; accessed on 11 April 2017) and PHOSIDA (http://phosida.de/; accessed on 18 May 2017).

Protein kinases and phosphatases were identified and classified using iTAK (http://bioinfo.bti.cornell.edu/tool/itak, accessed on 12 February 2018) and followed the KinBase nomenclature (http://kinase.com/web/current/kinbase/; accessed on 22 February 2018) [31].

Protein and phosphoprotein cellular localization and molecular functions were determined using TAIR GO classification (https://www.arabidopsis.org/tools/bulk/go, accessed on 10 May 2022). Biological processes classification was determined with Classification SuperViewer (https://bar.utoronto.ca/ntools/cgi-bin/ntools_classification_superviewer.cgi; accessed from 17 May 2017).

The distribution of proteins and phosphoproteins in the Seednet network was analyzed in Cytoscape after importing network data available online (www.vseed.nottingham.ac.uk; accessed on 8 October 2019 [32]).

GO term enrichments were determined in Cytoscape using ClueGO application (pV < 0.01; hypergeometric test with Bonferroni pV correction) [33].

Phosphoprotein networks were constructed in Cytoscape from interaction data retrieved from STRING 11.0 (https://string-db.org; accessed from 6 May 2020), with a confidence cut-off over 0.4. MAPMAN classification was subsequently used for functional association [82].

## 5. Conclusions

As a whole, this study provides a first detailed view of protein phosphorylation changes in relation to seed germination capacity in *Arabidopsis* and highlights a deep remodeling of seed phosphoproteome in germinating seeds. It sheds light on the existence of an early and massive wave of protein dephosphorylation that affects a common pool of phosphoproteins independently of the germination capacity of seeds but also targets specific subsets of phosphoproteins in germinating and non-germinating seeds. In addition to this initial massive reset of seed phosphoproteome, de novo protein phosphorylation occurs, is restricted to germinating seeds, and precedes the emergence of the radicle. The existence of these different waves of protein dephosphorylation/phosphorylation and their correlation with seed germination suggest multiple functions for protein phosphorylation and its participation in both dry-to-imbibed transition and germination processes. A critical step forward will now be to unravel if and how these dynamic changes in phosphorylation status may participate in the go-no-go decision for seed germination.

## Data Availability

All the proteomic data are available by ProteomeXchange under the identifier PXD033347.

## Supplementary Materials

The following supporting information can be downloaded at: https://www.mdpi.com/article/10.3390/ijms23137059/s1.
