# Supplementary material for: Dynamics of Protein Phosphorylation during Arabidopsis Seed Germination
Source: Int J Mol Sci. 2022 Jun 24;23(13):7059. doi: 10.3390/ijms23137059 (PMC9266807; doi:10.3390/ijms23137059)
Supplement: Supplementary file 1 [file ijms-23-07059-s001.zip › Table_S1.pdf]

Table S1. Dimethyl labels design

Triplex number	Light +28	Intermediate +32	Heavy +36	Rep number
Tx1	Internal standard 1	DI_dry_R1	LI_dry_R1	rep1
Tx2	Internal standard 2	DI_dry_R2	LI_dry_R2	rep2
Tx3	Internal standard 3	LI_dry_R3	DI_dry_R3	rep3
Tx4	Internal standard 4	DI_6_R1	LI_6_R1	rep1
Tx5	Internal standard 5	DI_6_R2	LI_6_R2	rep2
Tx6	Internal standard 6	LI_6_R3	DI_6_R3	rep3
Tx7	Internal standard 7	DI_16_R1	LI_16_R1	rep1
Tx8	Internal standard 8	DI_16_R2	LI_16_R2	rep2
Tx9	Internal standard 9	LI_16_R3	DI_16_R3	rep3
Tx10	Internal standard 10	DI_24_R1	LI_24_R1	rep1
Tx11	Internal standard 11	DI_24_R2	LI_24_R2	rep2
Tx12	Internal standard 12	LI_24_R3	DI_24_R3	rep3
